# The fate of the fresh autologous pericardium after right ventricular outflow tract reconstruction

**DOI:** 10.34172/jcvtr.2022.06

**Published:** 2022-03-07

**Authors:** Hassan Tatari, Gholamreza Omrani, Maedeh Arabian, Kambiz Mozaffari, Yaser Toloueitabar, Sanaz Asadian, Nader Givtaj, Maziar Gholampour Dehaki, Amirhosein Jalali

**Affiliations:** ^1^Rajaie Cardiovascular Medical and Research Center, Iran University of Medical Sciences, Tehran, Iran

**Keywords:** Autologous Pericardium, Right Ventricular Outflow Tract (RVOT) Reconstruct, Histopathology

## Abstract

***Introduction:*** The autologous pericardium, treated or fresh, is used in reconstructive cardiovascular surgery. We aimed to describe the features of fresh pericardium utilized in right ventricular outflow tract (RVOT) reconstruction, years after the initial surgery.

***Methods:*** This cross-sectional study was performed on 72 patients (65.3% male, mean age =18.68 ± 9.63 y) with a history of RVOT reconstruction with the fresh autologous pericardium who underwent reoperation. During the surgery, a 1 × 1 cm sample was cut from the previous pericardial patch, and hematoxylin and eosin (H & E), Masson’s trichrome, and immunohistochemistry (IHC) staining was conducted. All the stained slides were evaluated,and the descriptive results were explained.

***Results:*** The mean follow-up duration was 13.48 ± 7.38 years. In preoperative evaluations,53 (73.6%) patients exhibited no RVOT dilatation, 17 (23.6%) showed mild RVOT dilatation,and 2 (2.8%) had RVOT aneurysms. The H & E staining revealed no calcification in 80.55%(58/72), mild calcification in 9.72% (7/72), and moderate calcification in 9.72% (7/72) of the total samples. None of the specimens demonstrated a marked calcification. All the samples were positive for CD31, CD34, smooth muscle alpha-actin, and von Willebrand factor in IHC. In Masson’s trichrome staining, on average, 64.74% (±18.61) of the tissue sections contained collagen fibers.

***Conclusion:*** The fresh autologous pericardium, utilized for RVOT reconstruction, showed viability, growth potential, positivity for endothelial cell markers, vascular differentiation,insignificant calcification, and no stenosis at long-term follow-up. We would, therefore, suggest it as a suitable choice for such reconstructive operations. Moreover, its usage during total correction of tetralogy of Fallot could be safe, feasible, and durable.

## Introduction

Both natural and artificial materials are utilized for reconstructive purposes in the field of congenital cardiovascular surgery. A tissue widely applied in this setting is the pericardium. It is applied in 3 types: xenografts (animal graft), allografts (homograft), and autografts. Of these, the autologous pericardium is the most popular because of its inexpensiveness and availability. While advantages and disadvantages are described for each type in some studies, the selection of the best choice for each procedure is an issue of controversy among expert surgeons of the field. ^1^ For instance, for all the advantages of the autologous pericardium in either processed (treated) or fresh (untreated) forms enumerated by a considerable number of studies, the superiority of either of these 2 types has yet to be well established. ^2-4^

Proponents of autologous fresh pericardium believe that not only does it reduce the development of future pericardial calcification but also it increases the chance of live pericardial cells to survive, which ensures commensuration with the growth of the patient and greatly lessens the likelihood of patch-site stenosis.^5^ In contrast, some experts prefer the treated autologous pericardium, which is prepared by exposure to glutaraldehyde, and posit that glutaraldehyde-treated patches are easier to handle in limited anatomic exposures and the strength of the cross-links between glutaraldehyde and collagen boost the strength of the pericardial patch.^3-6^

In this investigation, based on the hypothesis that distinct histologic characteristics provide a guide for the selection between fresh and treated pericardium in reconstructive surgery, we describe the histopathological features of untreated pericardial patches for right ventricular outflow tract (RVOT) reconstruction utilized many years previously.

## Materials and Methods

### Study population

This descriptive single-center cross-sectional study enrolled 72 patients (65.3% male, mean age = 18.68 ± 9.63 y) who had undergone RVOT reconstructive surgery with the fresh (non-treated) autologous pericardium and who were candidates for reoperation. Trans-annular patches had been utilized for all of the study subjects in their previous operation based on available operative notes. A comprehensive survey of medical records, operative notes, and echocardiographic findings was performed. Demographic data, consisting of age, sex, presence of comorbidities, medications, the primary diagnosis, the type of previous surgery, the indication of the reoperation, the interval between the 2 surgeries, the type of the current surgery, the presence of RV dysfunction, and the severity of tricuspid regurgitation, were recorded.

All the individuals recruited in the study signed informed consent forms for pericardial patch sampling before surgery. This study was approved by the institutional Ethics Committee.

### Right ventricular outflow tract diameter measurement

All of the patients had a cardiac MRI before their reoperation based on the institutional protocols in our center. Therefore, RVOT diameter was measured by an expert radiologist. The RVOT diastolic anteroposterior diameter was compared with that of the aortic annulus and the patients were classified as follows:

1. Normal RVOT diameter: RVOT diameter is equal to or less than the aortic annulus diameter.
2. RVOT dilatation: RVOT diameter is greater, but not 1.5 times, than the aortic annulus diameter.
3. RVOT aneurysm: RVOT diameter is at least 1.5 times greater than the aortic annulus diameter.

### Pericardial patch sampling

After the application of cardiopulmonary bypass, injection of the cardioplegic solution, and induction of cardiac arrest, a small sample (≈ 1 × 1 cm) was cut from the middle part of the previous pericardial patch. The sample was thereafter placed in the formalin solution (10%) and sent to the pathology department for further assessment.

### Histologic study

We assessed the presence of calcification, collagen fibers, and viability/differentiation in the samples utilizing hematoxylin and eosin (H & E), Masson’s trichrome, and immunohistochemistry (IHC) staining, respectively.

#### Hematoxylin and eosin (H & E) staining

**Figure 1:**
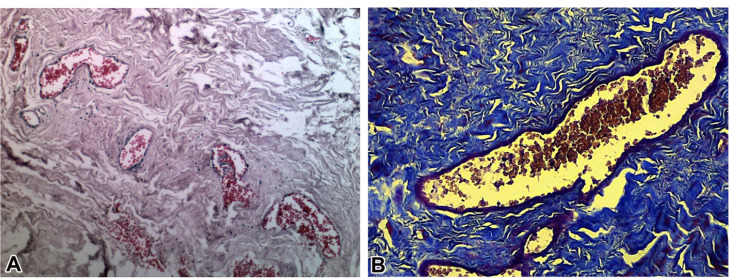
Two images obtained on pericardium sections are stained using hematoxylin and eosin (A) or Masson's trichrome (B). Hematoxylin and eosin section shows fibroconnective tissue with angiogenesis. In the Masson's trichrome staining, collagen-rich regions are colored in blue and Connective Tissue, Muscle and, Epithelium appear in red and purple.

The explanted tissue was investigated for gross calcification via H & E staining after it was dehydrated, embedded in paraffin, and cut into 5 mm sections. All the sections were evaluated by an experienced pathologist. The reports were registered as none, minimal, mild, moderate, and marked calcification based on the subjective estimation of the pathologist (Figure 1).

#### Masson’s trichrome staining

This technique depicts connective tissue compartments, mainly collagen. As is denoted by its name, this method is a 3-color protocol whereby nuclei will be blue; collagen will be green or blue; and cytoplasm, keratin, muscles, and red blood cells will become red. The procedure was performed by the immersion of the fixated sample into iron hematoxylin and 3 well-prepared solutions. The light microscope was applied to detect the Collagen fibers in the pericardial samples, with 400×magnification (Olympus, Hamburg, Germany). Image J software was utilized for the analysis of the stained slides (Figure 1).

#### Immunohistochemistry (IHC) staining

**Figure 2:**
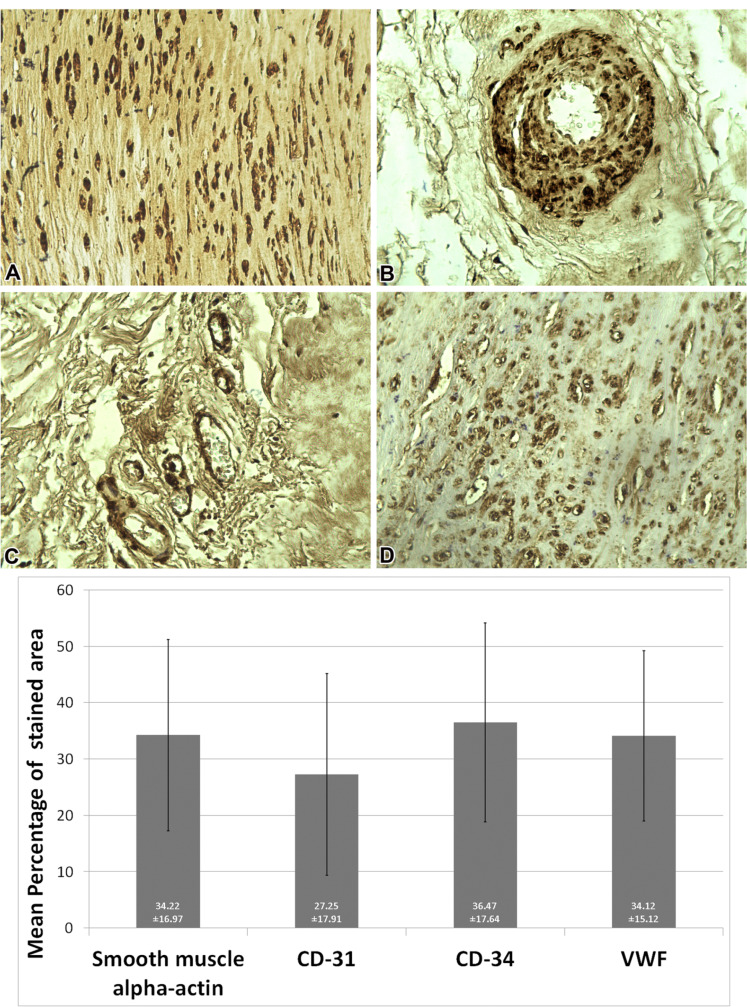
Representative pictures of the pericardium sections and quantitative analysis. Above, immunohistochemical staining of smooth muscle alpha-actin (A), a normal muscular artery (B), a few (normal number) venules in acceptable limits (C), and VWF (D). Below, quantification of the stained area. Data are expressed as mean ± standard deviation.

This unique procedure selectively identifies the proteins of the tissue cells based on the principle of the binding of an antibody (Ab) to an antigen (Ag). ^7^ The tissues, which were embedded in paraffin, were sectioned with a microtome at a range of 5 to 7 µm. In the next step, the sections were fixed on slides and dehydrated with the use of alcohol. The slides were cleared before the acquisition of microscopic images. The process can be carried out either directly by utilizing an Ab against a certain Ag or indirectly in a 2- or 3-step protocol. ^8^ In our research, a 2-step indirect procedure was performed. Horseradish peroxidase (HRP) enzyme was used to catalyze a color-producing reaction between Ags and Abs. Primary Abs against cell markers, consisting of CD34 (stem cell and endothelial cell marker), CD31, smooth muscle alpha-actin, and von Willebrand factor (VWF) (endothelial cell marker), as well as secondary HRP-bound Abs, were applied to determine the positivity of the samples for the mentioned markers. The light microscope was applied to detect the positive CD markers with 400×magnification (Olympus, Hamburg, Germany), and images were analysed with Image J software (Figure 2).

### Statistical analysis

IBM SPSS Statistics, version 22, was employed for the statistical analyses. According to the descriptive nature of the study, the results were depicted in tables. Categorical variables were represented as frequencies and percentages, while continuous variables were described as the mean ± the standard deviation or the median in terms of the normality of the distribution. One-way ANOVA was applied to investigate the difference in mean follow-up time between the five groups of patients regarding calcification.

## Results

The present study was conducted on 72 patients (65.3% male, mean age = 18.68 ± 9.63 y). The youngest patient was 2 years old, and the oldest was 43 years of age. The primary diagnosis was tetralogy of Fallot in 98.6% (71/72) of the patients. One patient was diagnosed with ventricular septal defect (VSD) in combination with pulmonary stenosis. No medical comorbidity was observed in the study population. In all the patients, a fresh (untreated) autologous pericardial patch had been used in their previous surgery to correct RVOT and the main pulmonary artery. The mean follow-up duration was 13.48 ± 7.38 years. The minimum and maximum intervals between the 2 operations were 1 and 35 years, respectively. The most frequent indication for reoperation was severe pulmonary insufficiency (88.9%, 64/72). In preoperative cardiac magnetic resonance evaluations, 53 (73.6%) patients revealed no RVOT patch dilatation, 17 (23.6%) exhibited mild RVOT patch dilatation, and 2 (2.8%) had an aneurysm at the RVOT patch site. On MRI, all of the RVOT patches revealed late gadolinium enhancement (post-operative changes). Table 1 demonstrates the demographic data of the study population.

Intraoperative assessment of the surgeons declared soft consistency with smooth internal and rough external surfaces of the RVOT pericardial patches. No obvious calcification was detected. The patches appeared to be dilated in 11/72 (15.27%) and aneurysmal in 2/72 (2.77%) of the patients.

**Table 1:** Demographics and clinical data of the study population

**Variables**	**Mean±SD**
Age	18.68 ± 9.63
Follow-up time	13.48 ± 7.38
Variables	**Frequency**
Gender	
Male	47 (65.3%)
Female	25 (34.7%)
Primary Diagnosis	
TOF	71 (98.6%)
VSD + PS	1 (1.4%)
Previous Operation	
TFTC	69 (97.2%)
TFTC +PVR	1(1.4%)
VSD closure + PS repair	1 (1.4%)
Size of RVOT Aneurysm	
No RVOT patch dilatation	53 (73.6%)
RVOT patch dilatation	17 (23.6%)
RVOT patch aneurysm	2 (2.8%)
TR Severity	
Mild	39 (54.2%)
Moderate	30 (42.3%)
Severe	2 (2.8%)
RV Dysfunction	
Mild	11 (15.3%)
Moderate	59 (81.9%)
Severe	2 (2.8%)

Abbreviations: TOF, tetralogy of fallot; VSD, Ventricular septal defect; PS, pulmonary stenosis; TFTC, tetralogy of fallot total correction; PVR, pulmonary valve replacement; RVOT, right ventricular outflow tract; TR, tricuspid regurgitation

The H & E staining revealed no calcification in 80.55% (58/72), mild calcification in 9.72% (7/72), and moderate calcification in 9.72% (7/72) of the total samples. None of the examined samples demonstrated marked calcification. Moreover, the difference in mean follow-up time between our five calcification groups was not significant (*P* = 0.5).

The IHC analysis confirmed the positivity of all the examined samples for all the evaluated markers, comprised of CD31, CD34, smooth muscle alpha-actin, and VWF. On average, of the cut surfaces of all the samples, 36.47% ( ± 17.64) were positive for CD34, 34.22% ( ± 16.97) for smooth muscle alpha-actin, 34.12% ( ± 15.12) for VWF, and 27.25% ( ± 17.91) for CD31 (Figure. 2). In Masson’s trichrome staining, on average, 64.74% ( ± 18.61) of the tissue sections contained collagen fibers (analysis via Image J software). The results of the histopathologic studies are summarized in Table 2.

**Table 2:** Results of the histopathologic studies

**Calcification in the Pericardial Patch**	
None	(58/72) 80.55%
Minimal	(0) 0%
Mild	(7/72) 9.72%
Moderate	(7/72) 9.72%
Marked	(0) 0%
Stained Area for IHC Markers	
Smooth muscle alpha-actin	34.22% ( ± 16.97)
CD-31	27.25% ( ± 17.91)
CD-34	36.47% ( ± 17.64)
VWF	34.12% ( ± 15.12)
Masson’s Trichrome Staining	
Collagen fiber percentage of tissue sections	64.74% ( ± 18.61)

Abbreviations: IHC, immunohistochemistry

## Discussion

Corrective surgery in congenital heart diseases is performed with the aid of an array of biological and prosthetic materials. All synthetic and most biological products have fixed dimensions and cannot grow over time, thereby inevitably narrowing the bloodstream passage as the patient grows up.

The utilization of a substance with the potential of remaining viable over time in reparative operations would be allied with much fewer complications. The pericardium is a unique available structure that has been used for many years in the field of congenital cardiac surgery on the basis of the assumption that it possesses proliferative and differentiative capabilities.

We investigated the histopathologic features of the fresh pericardial patch utilized for RVOT reconstruction in the setting of tetralogy of Fallot total correction a long time after the initial surgery. The main findings of our interrogation are as follows:

All of our samples revealed viability and endothelial cell markers as documented by the positivity for CD31, CD34, VWF, and smooth muscle alpha-actin. Preponderantly, our study subjects revealed no calcification and remarkably, none of the cases showed marked calcification. Our studied samples showed positivity for collagen, with a high percentage of slide surfaces revealing non-degraded collagen. Aneurysmal dilatation was detected just in 2/72 of our cases (based on the pre-operative cardiac MRI measurements), while none of them exhibited stenosis.

In 1984, Guyton et al concluded that broadly based autologous pericardial flaps possessed growth potential and hypothesized that such flaps could be appropriate for the repair of pulmonary arteries. ^9^ Shortly after their valuable investigation, Hvass et al reported remarkable results pertaining to 3 cases operated on with pedicled pericardium for pulmonary artery hypoplasia repair and stated that follow-up angiography and echocardiography confirmed a uniform increase in the size of the reconstructed pulmonary arteries after a period of 10 months to 2.5 years. ^10^ Similarly, Khoury et al reported excellent results in a 6-year follow-up of patients who underwent RVOT or pulmonary artery repair utilizing pedicled pericardial patches and demonstrated the growth of the pedicled pericardium in parallel to that of the pulmonary arteries. ^11^ Such findings reinforce the concept of the application of the living tissue likely due to the preserved growth and repair potential in such tissues, which may reduce the frequency of the occurrence of stenosis or degenerative changes over time as the patient grows up. ^12^ In our study, we observed that the untreated pericardial patch maintained its viability and grew appropriately as evidenced by the positivity for viable cell markers and the absence of RVOT stenosis in our long-term follow-up, respectively.

Our microscopic evaluation (H & E staining) revealed a 3-layer configuration for the pericardium: the serosa, the fibrosa, and an external layer of connective tissue. The serosa is composed of mesothelial cells. The fibrosa is the layer of compact collagen bunches with few elastic fibers between them, and the outer layer is rich in elastic fibers, adipose tissue, neural components, and blood vessels. The normal mesothelial cell is negative for CD31, CD34, VWF, and smooth muscle alpha-actin.^13-15^ Strikingly, our investigation revealed positive endothelial cell markers and smooth muscle presence in all the pericardial samples. Therefore, chiming in with the research by Hibino et al, we assume that the pericardial tissue not only preserves growth potential but also morphs into vascular tissue.^13^

Hodges et al posited that the autologous pericardium could provide optimal functional outcomes at long-term follow-up. The slow process of calcification and the lesser likelihood of immune response are the main stated advantages of the autologous pericardium. ^16^ Fukunaga et al reported a case of mitral restenosis 5 years after an initial aortic and mitral valve repair surgery with the glutaraldehyde-treated autologous pericardium. ^17^ Our study strongly supports the idea of less late calcification development in the application of the fresh pericardial patch in comparison with the glutaraldehyde-treated pericardium. Another noteworthy point is that the use of such non-fresh pericardial tissues as the glutaraldehyde-treated autologous pericardium, the allograft, and the animal pericardium is accompanied by severe calcification, which may complicate possible re-interventions with technical problems in reconstructive procedures or artificial valve implantation. Prolonged procedure times and debridement requirements are instances of such complications, which could even result in the further destruction of the adjacent tissue and postoperative bleeding.

López Marco et al in their case report of aneurysmal dilatation late after supravalvular aortic stenosis repair with the fresh autologous pericardium, emphasized the inappropriateness of its use in the systemic circulation or RVOT given the probability of distal stenosis. They recommended the fixation of the autologous pericardium with glutaraldehyde before implantation in such situations so as to augment tensile strength and lessen the consequent stretching of the patch and the risk of aneurysm formation. ^12^ In another case report, the authors described a neonate with VSD as a part of a more complex congenital anomaly who underwent VSD closure with the fresh autologous pericardium and pulmonary artery reconstruction. The case was complicated and was referred for reoperation 4 months later due to an aneurysmal pericardial patch at the site of the VSD repair. The authors, albeit uncertain, explained multiple probable mechanisms to clarify the complication. ^18^ One hypothesis underscores the degeneration of the patch as a possible trigger of aneurysmal dilatation as evidenced by the loss of collagen fibers. Our study revealed an adequate amount of collagen in all extracted samples, which implies the acceptable strength of the patch after a long period. The findings are in line with a recent investigation on the outcome of the fresh autologous pericardium utilized for mitral leaflet repair insofar as it concluded that the untreated autologous pericardium was excellent for sophisticated mitral valve leaflet repair without evidence of late patch aneurysmal formation. ^19^

In our research, the diagnosis of RVOT patch dilatation and aneurysm was made based on the preoperative cardiac MRI measurements. These measurements were different to some extent with the intraoperative visual assessment of the surgeons regarding mild dilatation, but the two patients who were considered to have RVOT patch aneurysm on preoperative MRI were diagnosed precisely by the surgeons. The low incidence of aneurysmal dilatation of the RVOT patch could be further confirmed knowing the fact that an anterior augmentation of RVOT with Dacron or Bovine pericardial patch needed to be performed in all of the patients for proper placement of prosthetic pulmonary valve, except the two patients with aneurysmal previous RVOT patch. Accordingly, the patches had acceptable configuration at the end of surgery.

Contrary to the notion that glutaraldehyde bolsters the strength of the patch, an animal study on the thoracic aortic patch conducted by Haluck et al demonstrated that glutaraldehyde not only failed to append any strength to the patch but also diminished the expansile potential of the graft in comparison with the fresh pericardium.^20^ Further, this type of pericardium lacks viable cells, fails to grow in tandem with the patient, and is associated with a high prevalence of calcification. The debate on the superiority of different patch types, however, continues.

In light of our findings, we believe that a comparison between the pericardium utilized in the previous surgery and the intact pericardium could confer more reliable results. Nevertheless, the fact that a part of the pericardium is used in the previous operation and a part of it may be required in reoperation renders the extraction of an adequate sample somehow difficult. Needless to say, further large-scale multicentric investigations with the potential of comparing the histopathologic fate of different patch types in different anatomic settings will yield more robust results.

## Conclusion

We concluded that the fresh untreated autologous pericardium utilized for RVOT reconstruction showed viability, growth potential, positive endothelial cell markers, vascular differentiation, insignificant calcification, and no stenosis at long-term follow-up. Considering all these advantages and its inexpensiveness, the fresh untreated autologous pericardium can be deemed a worthy, if not the first, choice for such reconstructive surgeries.

## Acknowledgements

We want to thank Ms. Yasaman Jahandideh for her kind assistance in the data gathering.

## Funding

No funding was achieved for this study.

## Ethical approval

This study is approved by the institutional and country ethic committee with the following approval code: RHC.AC.IR.1395.23

## Competing interests

Authors have nothing to disclose with regard to commercial support.
